# Current and emerging approaches to cochlear immunosuppression with translation to human inner ear stem cell therapy: A systematic review

**DOI:** 10.1371/journal.pone.0318165

**Published:** 2025-02-13

**Authors:** Nathan J. Creber, Jameel Muzaffar, Shravan Gowrishankar, Daniele Borsetto, Veronica Phillips, Matthew E. Smith

**Affiliations:** 1 Cambridge University Hospitals NHS Trust, United Kingdom; 2 Institute of Academic Surgery, Royal Prince Alfred Hospital, Sydney, Australia; 3 University of Cambridge, United Kingdom; Sapienza University of Rome: Universita degli Studi di Roma La Sapienza, ITALY

## Abstract

Hearing loss is a significant health burden across all stages of life. One in 5 people suffer hearing loss, with 5% of the world’s population experiencing disabling hearing loss. A large proportion of this loss is the consequence of damage or loss of neurosensory structures, termed “sensorineural” hearing loss. A recent advance in the treatment of sensorineural hearing loss has occurred, with the advent of inner ear stem cell therapy. Focus has pivoted from augmenting existing neural structures to regenerating neural frameworks. To date, stem cell therapy is limited by the host immune system and rejection of donor cells. A better understanding of immunity in the inner is ear required to progress stem cell therapy for hearing loss. This review outlines a contemporary understanding of the inner ear immune system. We discuss concepts of immune dysregulation that may lead to common inner ear pathologies, and, in doing so, review the efficacy of current pharmacotherapies that mitigate end organ damage through a process of immunosuppression. Current literature is appraised through a systematic review exploring two areas of focus; immunosuppression therapies for the treatment of inner ear pathology associated immune dysregulation, and, subsequently, the efficacy of immunosuppressive agents in translational models of inner ear stem cell therapy. Through greater understanding of these concepts, and systematic appraisal of the literature, this review summarises the literature for contemporary immunosuppressive regimes that may facilitate stem cell accommodation in the cochlea and auditory nerve.

## Introduction

Hearing loss is a significant health burden across all stages of life. Currently approximately 420 million people are affected by disabling hearing loss worldwide, accounting for over 5% of the world’s population [1]. This figure is expected to grow to over 700 million people by 2050 [1]. A significant proportion can be avoided though preventive health measures, with approximately 60% of childhood hearing loss being attributed to preventative issues [1]. This leaves a substantial and growing remaining proportion of our population requiring hearing rehabilitation. A large majority of these individuals are impacted by “sensorineural” hearing loss; characterised by a loss in structure or function of the neurosensory elements and/or neural pathways of the inner ear. Traditionally, therapies have focused on augmenting existing neural structures, either though amplification with hearing aids, or direct stimulation with implantable devices. More recently, neural regeneration therapies with stem cells have received wide attention for the treatment of neurodegenerative disorders, such as spinal cord injury and macular degeneration [2–4]. In a significant paradigm shift, the applicability of stem cell therapy is rapidly evolving to address neural deficits in similar organs and functions, such as the ear and hearing [5] with a pivot from neural augmentation to neural regeneration.

Current therapeutic approaches to stem cell transplantation have been hindered by limitations, including the method of delivery to the organ of corti within the cochlea, and adequate differentiation of stem cells to viable hair cells. As we overcome these limitations [6], and currently move towards commencing clinical trials, the issue of rejection of donor cells by the host immune system needs to be addressed. In recent events, the authors of this article have been approached by the biotechnology industry to offer their insights and recommendations concerning this. To address this, immunosuppression strategies have been extensively studied in retinal and spinal tissues. With respect to hearing therapy, lessons may also be acquired by appraising the current medical therapies for pathologies of the inner ear associated with immune dysregulation. This review outlines the contemporary understanding of the inner ear immune system. We discuss the concepts in immune dysregulation that can lead to common inner ear pathologies, recently termed “immune mediated inner ear disease” (IMIED), and in doing so review the current pharmacotherapies that aim to mitigate end organ damage through a process of immunosuppression. The current literature is appraised through a systematic review exploring two primary areas of interest. Firstly, an initial set of outcomes are presented exploring immunosuppression therapies for the treatment of inner ear pathology associated immune dysregulation, and secondly, a subsequent outcome explores the efficacy of immunosuppressive agents in translational models of inner ear stem cell therapy. Through an understanding of these concepts this review proposes immunosuppressive regimes that may facilitate stem cell accommodation in the cochlea and auditory nerve.

## Methods

The databases Medline, Embase, Scopus, Web of Science, and the Cochrane Library were searched from inception to March 2024, individually. Exact search terms used on each database (and each platform) are provided in the supplementary data (S1 File). The search strategy was formulated by a medical librarian using the PRESS checklist and evaluated against the PRISMA-S guidelines [7, 8]. Databases were searched separately, rather than multiple databases being searched simultaneously on the same platform. The search syntax was adapted for each database, and to account for variation between thesaurus terms/controlled vocabulary across each database. Results were deduplicated using Endnote 20 software. Endnote was set to identify articles as duplicates if they matched in the Author, Year, Title, Short Title, and Reference Type fields, and was also set to ignore differences in item record spacing and punctuation in these fields when identifying duplicates. Three authors (NC, JM, and MS) independently screened all titles and abstracts generated by the search and evaluated the full texts of relevant articles identified against the inclusion criteria. Any disagreement between the assessors on the suitability of articles for inclusion tackled by thorough discussion between assessors, or failing this, by referral to the senior author (MS). Studies were included in the review if they met the following criteria:

Outcome 1 (preliminary review):

1) Human clinical study data on therapeutic strategies for one of the following inner ear pathologies: Autoimmune inner ear disease, sudden sensorineural hearing loss, Meniere’s disease, or cochlear implantation.
2) Detailed the immunosuppression regime used.

Outcome 2 (primary systematic review):

3) Reported data on stem cell implantation and survival, with detailed immunosuppressive regimes, in human or translational animal studies of inner ear stem cell therapy.
4) Detailed the immunosuppression regime used. (or specifically detailed the absence of immunosuppression).

Non-English studies were excluded. Studies containing aggregated data or duplicated data from previously published work were excluded, as were review articles, case reports, editorials, and letters. There were no limits set on publication year. Data were extracted by authors MES and NJC, in January 2023 and May 2024. The limited extraction did not highlight missing data. A list of all identified studies is included in the supplementary data (S2 File).

Primary outcome descriptor:

1. A systematic review of the efficacy of immunosuppression therapies for inner ear stem cell survival in human or animal studies.

### Risk of experimental bias

Risks of bias in individual studies included in the primary outcome descriptor were assessed independently by two separate reviewers (NC and DB) using the Systematic Review Centre for Laboratory Animal Experimentation (SYRCLE) risk of bias tool [9], disagreements again resolved by consensus.

## Results

### Outcome 1: Human clinical studies of immunosuppression for inner ear pathology

**Fig 1:**
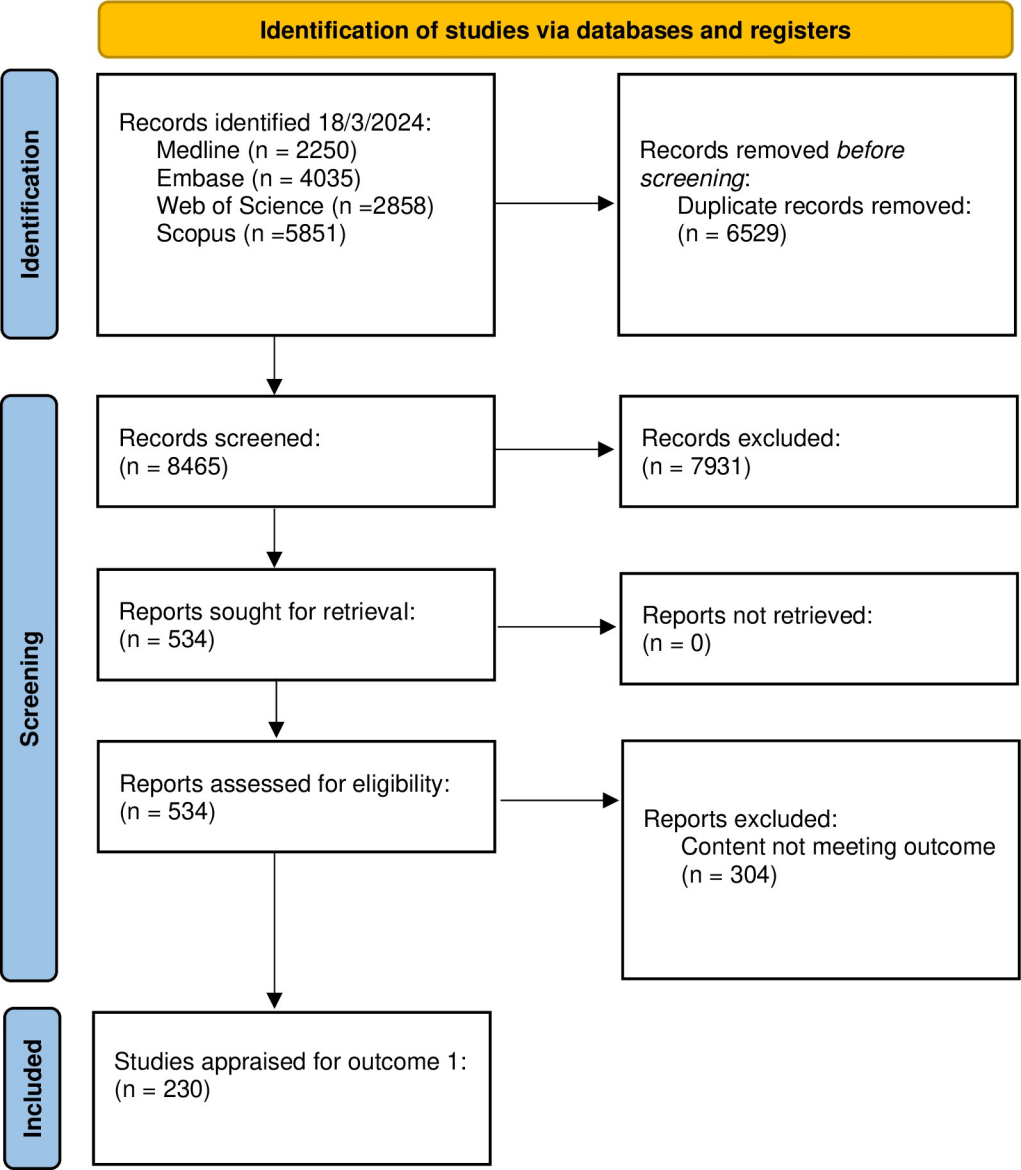
PRISMA flow diagram for identification and screening of reports for outcome 1.

A literature search yielded 14,994 papers. From reviewing title and abstract, 14,460 articles were excluded as not meeting criteria or duplicated studies. On further author review of the remaining 534 articles, 304 articles were excluded from narrative review based on abstract content, with 230 full texts assessed (Fig 1).

There are various conditions for which inner ear immunosuppression has been proposed as primary treatment, although all these disorders have weaknesses as models for immunosuppression relevant to stem cell transplantation. Immunosuppression of the inner ear has been used primarily in the treatment of autoimmune ear disease, sudden sensorineural hearing loss, Menieres disease, and to reduce inflammation at the time of cochlear implantation. Together these applications provide the only human clinical data on the effect immunosuppressive agents have on the inner ear, but the aetiology of many of these conditions is poorly understood and may not reflect inflammation, meaning clinical outcomes cannot be directly extrapolated as a marker of inner ear immunosuppression. A natural history of resolution for many conditions and primarily retrospective data series further hinder interpretation of data.

For the above reasons, human clinical data on inner ear immunosuppression is primarily of use in comparing the relative effects of different immunosuppressive agents and their route of administration, rather than absolute treatment outcomes. The section below summarises human clinical studies where a comparison has been directly made between different immunosuppressive regimens, with differences in outcome potentially indicating the efficacy of inner ear immunosuppression.

#### Autoimmune inner ear disease

Autoimmune inner ear disease (AIED) is a poorly defined condition, that may be primary, when the pathophysiology is limited to the ear itself. However it is estimated that 15–30% of AIED is not ear-specific and is instead part of a systemic autoimmune disease such as rheumatoid arthritis, systemic lupus erythematosus or Cogan syndrome [10]. There are likely many underlying pathologies, and it is worth noting that many cases do not respond to immunosuppression: in one study 65% of ears saw no change in their pure tone audiogram (PTA) (±5 dB) from baseline with steroid treatment [11]. To date no serological test has proven of significant value in defining AIED patients, and clinical diagnosis is typically made on the basis of a beneficial response to immunosuppressive therapy, and usually corticosteroids [12, 13].

There are several comprehensive reviews published that summarise the immunosuppressive agents that have been used for AIED [12–15]. Most data relate to small case series, and the variable populations and reported outcomes greatly limit the extent to which immunosuppression can be compared between agent or regimens.

The immunosuppressive agents used in identified studies for AIED are summarised in Table 1.

**Table 1:** Immunosuppressive agents in AIED.

Agent	Mechanism	Dose
*Systemic*		
Prednisolone	Corticosteroid (various effects)	60mg/day oral most common, (various others employed)
Methylprednisolone	Corticosteroid (various effects)	24mg to 1mg/kg, tapered
Methotrexate	Inhibition of purine biosynthesis Antagonism of folate, downregulation of T-cells	7.5mg increasing to 15–25 mg per week
Cyclophosphamide	inhibition of DNA replication, affects lymphocytes	100mg twice a day
Azathioprine	Inhibition of purine biosynthesis	100mg twice a day
Rituximab	AntiCD20 antibody	Various
Anakinra	IL-1 receptor antagonist	Various
Etanercept	TNFα inhibitor	Various
*Intratympanic*		
Dexamethasone	Corticosteroid (various effects)	4mg/ml to 24mg/ml
Methylprednisolone	Corticosteroid (various effects)	20mg/ml to 80mg/ml up to once per week for 2 months
Infliximab	TNFα inhibitor	Various
Golimumab	TNFα inhibitor	Various
Etanercept	TNFα inhibitor	Various

*Steroids*. Systemic steroids are often used as first line treatment for AIED, and have different activities that may benefit patients with AIED: anti-inflammatory, immunosuppressive, and anti-oedema effects. Immunosuppression reduces the formation of immune complexes, while anti-inflammatory and anti-oedema actions restore the normal calibre of affected vessels. Not all effects of steroids in AIED may therefore be relevant to immunosuppression within the labyrinth. The efficacy of oral steroids in improving hearing is reported to range from 50–70% [14] in initial treatment of AIED, though most patients become poorly responsive to steroid over time.

Steroids are commonly given by both systemic and intratympanic routes in clinical practice. However, the evidence for differences in route of administration is weak, and surpassed by work done in the field of sudden sensorineural hearing loss (detailed below).

*Disease-modifying antirheumatic drugs (DMARDs)*. Various DMARDs are used in the treatment of AIED, with cyclophosphamide and methotrexate most commonly used, and azathioprine and mycophenolate mofetil also trialled. The low number of studies for each intervention and significant heterogeneity of population, intervention and outcomes, has meant that meta analysis has been of limited benefit. Gordis *et al*. included 12 retrospective and prospective studies in a metanalysis of a disease-modifying antirheumatic drugs as treatment of autoimmune inner ear disease [16]. Treatment included 187 patients receiving heterogenous mix of interventions including methotrexate (5 studies) and biologics (4 studies). DMARDs demonstrated statistically, though not clinically significant improvement in pure tone thresholds and a moderate improvement in speech discrimination thresholds.

Little high-level evidence has been generated. In an RCT setting, methotrexate was no more effective than placebo in maintaining the hearing improvement attained with prednisone treatment in patients with inner ear autoimmune disease [17]. Similarly, in the only other identified RCT for AIED (a pilot study), etanercept was not found to be superior to placebo in improving pure tone average [18].

Intratympanic methotrexate is suggested to be safe, with middle ear mucosal oedema the only change identified in a rat model [19].

*Biologics*. A 2022 systematic review [20] summarised the evidence for biologics in inner ear autoimmune disease. The review identified data relating to agents targeting three molecular targets, tumour necrosis factor-alpha (TNF-α, thought to be produced by resident immune cells in the cochlea), CD20, and interleukin-1 (IL-1, a key regulator of local inflammation in the cochlea). These mediators are involved in the recruitment of immunocompetent cells to the cochlear [21]. Of 14 studies included, all had fewer than 23 participants, and with considerable heterogeneity in study population, interventions and outcomes, the effect of biologics in included studies were highly variable [20].

Animal models studies have been supportive of etanercept, a TNF blocker, in reducing inner ear inflammation. Satoh *et al*. found TNFα and IL-1b generated within the cochlea during surgical trauma and inflammatory insult [22]. Systemic etanercept reduced both the number of inflammatory infiltrating cells and cochlear fibrosis. Wang *et al*. employed intratympanic (via osmotic pump remote to the ear) and systemic etanercept in a guinea pig model of immune-mediated hearing loss [23]. Regardless of delivery route there was significantly less histologic evidence of inflammation in the cochlea from etanercept-treated animals, and reduced hearing loss. Etanercept was found not to be ototoxic, even with direct cochlea injection. Etanercept has also showed its equivalence to steroids in an induced labyrinthitis model [24].

Looking to the wider field of transplantation, monoclonal antibody (mAb)-based therapies have become a staple of induction and acute rejection therapy in solid organ transplant patients [25]. A series of mAbs have been developed to prevent transplant rejection. The mechanisms of these mAbs are diverse, but all target specific cluster designation (CD) proteins on the T or B cell surface. These include mAbs against CD3, CD25 and CD52.

*Route of immunosuppression administration in AIED*. Currently no clinical study has compared the clinical efficacy of oral and intratympanic steroids in AIED, and individual studies have significant heterogeneity of population and outcomes.

In a guinea pig model of keyhole limpet hemocyanin (KLH)- induced labyrinthitis, dexamethasone, ciclosporin, prednisolone acetate, fluorouracil and tacrolimus were delivered to the round window membrane via intratympanic injection or osmotic minipumps [26]. None of the immunosuppressants were effective at reducing hearing loss or inflammation, with no significant differenced found in histological analysis. This is in contrast to the above work by Wang *et al*. using a similar model with etanercept [23].

Local (e.g. intratympanic) administration of immunosuppression may be less relevant to AIED than when considering stem cell transplants due to extra-cochlear effects of the condition. As an example, with SLE several mechanisms for hearing loss are identified, with some occurring within the labyrinth (antibody/ antigen direct reactions, cytotoxic action) and others extra-cochlear such as immune complex deposition in auditory artery leading to hypoxia [27].

#### Sudden sensorineural hearing loss

The aetiology of sudden sensorineural hearing loss (SSNHL) is poorly understood, but inflammation is considered an important part of the pathophysiology in many cases, and the commonly used treatments for SSNHL in current clinical practice are designed to reduce inflammation. Although not a common condition, treatment outcomes for SSNHL have been well studied. Care must be taken when extrapolating clinical results as a marker of inner ear immunosuppression, as both the aetiology and the site of lesion in SSNHL is poorly understood, and some cases may represent lesions of the auditory nerve or other structures in the auditory pathway.

A Cochrane review explored the effect of intratympanic steroid on SSNHL [28]. Comparing intratympanic corticosteroids versus no treatment or placebo as secondary therapy, they found that intratympanic therapy may have a small effect on the change in hearing threshold, but appeared to be associated with a much higher proportion of participants whose hearing was improved (RR 5.55, 6 studies, low-certainty), and more favourable final hearing thresholds (mean difference -11.09 dB, 5 studies, low-certainty).

When Plontke *et al*. compared intratympanic corticosteroids versus systemic corticosteroids as primary therapy in their Cochrane review, they found no significant difference in any of; change in hearing threshold, the proportion of participants whose hearing was improved, or final hearing threshold (based on 7–14 studies, all conclusions of low-certainty).

The same Cochrane review compared intratympanic plus systemic corticosteroids (combined therapy) versus systemic corticosteroids alone as primary therapy. They found that combined therapy may have a small effect on the change in hearing threshold (mean difference -8.55 dB, low-certainty), but other benefits over monotherapy were very uncertain.

A systematic review and network meta-analysis published in 2019 [29] explored the outcome of different steroid therapies in SSNHL. They found that combination therapy, IT plus systemic steroids, demonstrated the largest difference in PTA improvement compared to placebo (25.85 dB, 95% CrI 7.18–40.58), followed by IV plus PO steroids (22.06 dB, 95% CrI 1.24–39.17), IT steroids (18.24 dB, 95% CrI 3.00–29.81) and finally PO steroids (14.7 dB, 95% CI 0.8–27.0). It was noted that many trials had unclear to high risk of bias.

Two systematic review-based studies by the same team have analysed several questions relevant to the technique and regimen for inner ear immunosuppression, exploring outcomes from primary [30] and secondary [31] treatment of SSNHL, utilising data from controlled and uncontrolled studies and mathematical modelling of cochlear drug levels. Intracochlear drug concentrations were calculated by a validated computer model of drug dispersion in the inner ear fluids based on the concentration and volume of glucocorticoids applied, the time drug remained in the middle ear, and on the specific timing of injections. 25 studies (28 treatment groups) were included in the secondary therapy review, 22 using dexamethasone, 6 using methylprednisolone. 30 studies (33 treatment groups) were included in the secondary therapy review, 19 using dexamethasone, 14 using methylprednisolone.

For primary and secondary intratympanic or combined therapy of ISSHL there was no dependence of change in hearing threshold (or the average final hearing threshold) on the drug used (dexamethasone or methylprednisonone), the concentration used, the number of injections, the frequency of injections, the estimated time of drug in the middle ear or the total duration of treatment.

Various explanations may account for these findings, including that steroid therapy may be ineffective for treating ISSHL, and spontaneous recovery is likely to mask some effects, thus highlighting the uncertainty with which the extent of immunosuppression can be extrapolated from outcomes in SSNHL data.

The agents, routes and doses of immunosuppression used in identified SSNHL studies and reviews is summarised in Table 2. No study used a non-steroid immunosuppression, though in some a non-immunosuppressant adjuvant was employed.

**Table 2:** Immunosuppressive agents in SSNHL.

Agent	Route	Dose
Dexamethasone	Intratympanic	3.3mg/ml to 24mg/ml
Methylprednisolone	Intratympanic	20mg/ml to 125mg/ml
Prednisolone	Oral	60mg or 1mg/kg max 80mg
Methylprednisolone	Oral	32mg-1 mg/kg
Prednisolone	Intravenous	60-250mg/day or 1 mg/kg//d
Methylprednisolone	Intravenous	500 mg/d
Dexamethasone	Intravenous	5–10 mg/d or 0.1mg/kg/d

*Meniere’s disease*. The aetiology of Menieres is not well understood, though steroids have proven efficiacy [32, 33]. Clinically employed immunosuppression for Menieres disease mirrors that of SSNHL, except where AIED is suspected. Only one study has directly compared local immunosuppressive treatments for Menieres disease in a randomised controlled trial [34], finding intratympanic methylprednisolone superior to intratympanic dexamethasone in improving hearing level, but not vertigo symptoms.

#### Cochlear implantation

Inflammation following cochlear implant electrode insertion trauma can be associated with poorer outcomes in patients, and has been a focus of translational and clinical research. The evidence in this field provides a potential marker of inner ear immunosuppression efficacy, and implant electrodes also provide a mechanism for intracochlear drug delivery. Studies may also be relevant as stem cell transplantation may necessitate low-level trauma from cochleostomy or round window injection.

As noted earlier in this report, Gay *et al*. have summarised preclinical and clinical evidence for reducing inflammation associated with CI, concluding that the use of glucocorticoids leads to reduced fibrosis within the cochlea, and reduced electrode electrical impedance following cochlear implantation surgery [35].

A recent randomised human study of high dose methylprednisolone vs placebo has suggested that there is no additional benefit with respect to hearing preservation, though all patients also received intravenous dexamethasone at induction of anaesthesia, which may explain this effect [36]. A further human study of a single high dose of systemic prednisolone did not provide significant reductions in post procedure impedances, when compared to controls. The same group performed a further human study in which triamcinolone was injected via an intracochlear catheter prior to cochlear implantation, with demonstrated protection of residual hearing, suggesting that this may be a suitable route for the administration of other drugs [37].

Directly comparing administration routes, a small randomised controlled trial by Kuthubutheen *et al*. compared perioperative oral and intratympanic steroid delivery to a control group [38]. Subjects receiving transtympanic steroid had better PTA thresholds (125 to 8000 Hz) at three and twelve months compared with the control and oral GC group, but no differences were found in speech discrimination.

Many of the issues around periprocedural administration of glucocorticoids are discussed further in the review by Fuentes *et al*. [39]. This summarises several systematic reviews and trials, with the conclusion that there appears to be short and possibly long-term benefits to the use of glucocorticoids at the time of implant surgery, but there is not clear superiority of oral, intratympanic or combined routes of delivery, in large part due to a lack of adequately designed trials to demonstrate this.

### Outcome 2: Translational models of stem cell therapy to the cochlea

**Fig 2:**
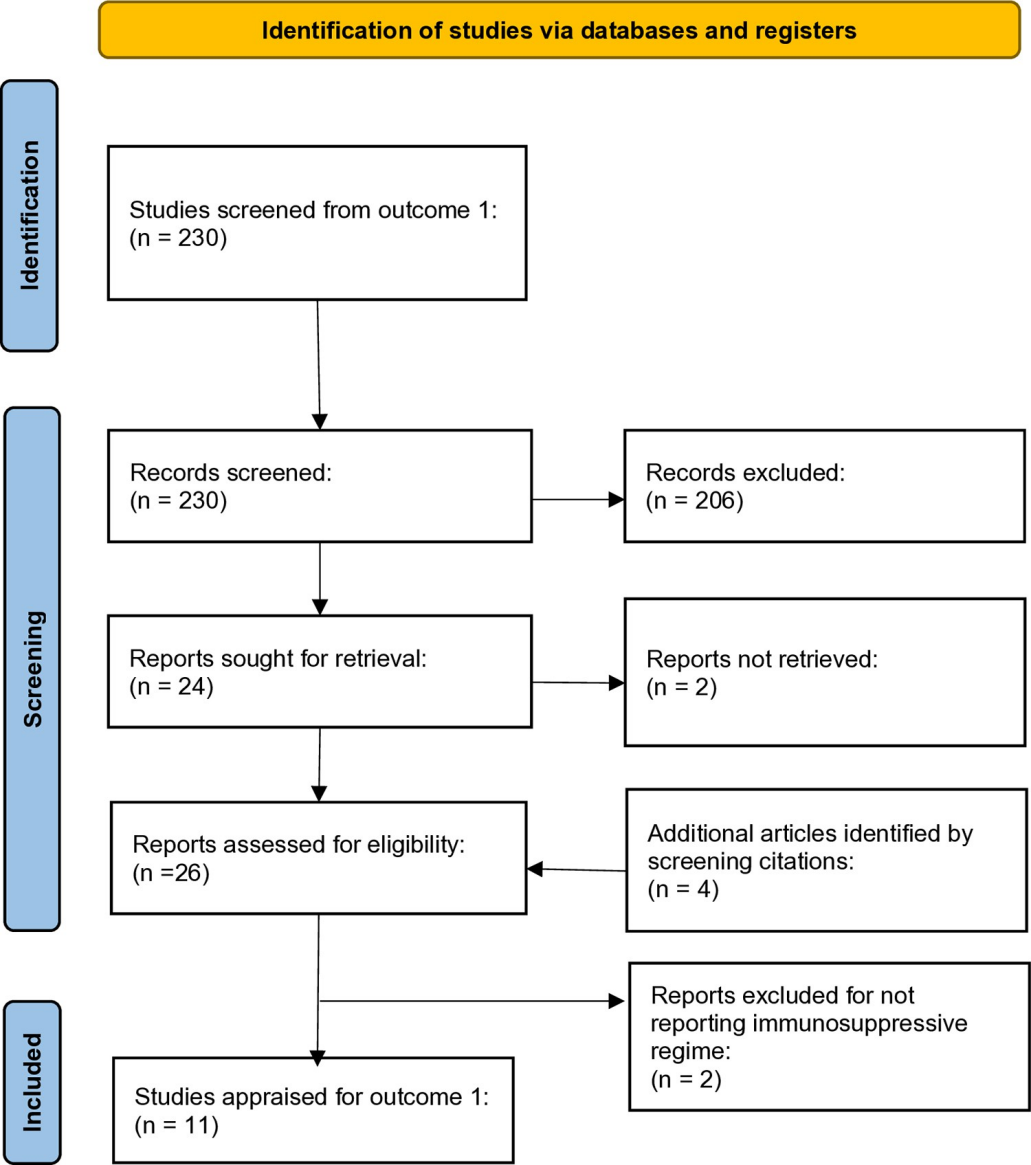
PRISMA flow diagram for identification and screening of reports for outcome 2.

Following abstract screening, of the 230 full texts assessed in search No.1, 24 articles proceeded to inclusion in outcome 2. For two articles no full text or further information was available, and data were extracted from the abstract. During subsequent full text review and data extraction, four further full text articles were identified as relevant via citations (Fig 2).

No articles were identified relating to human trials of stem cells for hearing loss, and all related to translational work in animal models. Articles that described either the presence or absence of immunosuppression were assessed for outcomes. 11 papers were identified (Table 3). 5 studies employed no immunosuppression, 6 used ciclosporin (US approved name ciclosporin) at a dose between 5.6 mg/kg/day (guinea pig) and 500mg/kg/day (mouse). There was considerable heterogeneity in the animal model, stem cells used, transplantation method and assessments. All studies employed a small mammal model as recipient (mouse, gerbil, rat or guinea pig). 10/11 studies utilised xenografts, 5 using human stem cells.

**Table 3:** Study characteristics of inner ear stem cell studies.

Author/Year	Model	Cell	Delivery	Immuno-suppressive agent	Immuno-suppression regimen	Findings
Hildebrand 2005 [40]	Guinea pig Aminoglycoside (kanamycin) deafened	Mouse Undifferentiated & partially differentiated embryonic SC	Intracochlear (needle via round window)	None	None	No evidence of hyperacute rejection, or acute rejection. At 9 weeks no significant inflammation, with no signs of immunological reactivity in the scala media.
Lang 2008 [41]	Gerbil Ouabain treated	Mouse Embryonic SC	Intracochlear (needle via round window—direct to Rothenthals canal or perilymphatic or endolymphatic injection)	Ciclosporin	15 mg/kg/day s.c., starting 1 day before surgery and terminating the day before sacrifice.	Viable clusters of SCs within Rosenthal’s canal and perilymphatic spaces appeared to be associated with neovascularization. A small number of SCs within Rosenthal’s canal stained for mature neuronal or glial cell markers. SCs introduced into perilymph survived in several locations, but most differentiated into glia-like cells. SCs transplanted into endolymph survived poorly if at all.
Cho 2011 [42]	Guinea pig Ouabain	Human Neural differentiated mesenchymal SC	Intracochlear, cochleostomy (scala tympani)	None	None	At 6 weeks there was no inflammatory response in grafted cochlea Number of neurons in each turn of the spiral ganglion was significantly greater in the stem cell group Human cells survived in the spiral ganglion and showed neuronal differentiation Transplanted ears had significantly better ABR
Zhang 2013 [43]	Rat Ouabain treated	Mouse Neural SC derived from olfactory bulb	Intracochlear, cochleostomy (scala tympani)	Ciclosporin	15 mg/kg/day SC	SCs could migrate to and survive in Rosenthal’s canal Some SCs differentiated into spiral ganglion cells within Rosenthal’s canal
Gokcan 2016 [44]	Rat 15/20 IPS-treated were amikacin deafened	Mouse Induced pluripotent SC derived from embryonic fibroblasts	Intracochlear, cochleostomy (scala tympani)	Ciclosporin	IM 15 mg/kg daily from preoperative day 2 until postoperative day 7	At 4–6 weeks there was no difference in ABR or hearing between SC treated and control
Barboza 2016 [45]	Guinea pig neomycin-injured	Mouse Inner ear SC	Intracochlear, cochleostomy (scala tympani)	None	None	No evidence of infiltration by inflammatory cells at 2 weeks after transplantation Some SCs were observed in all scalae of the basal turns of the injured cochleas A proportion of SCs expressed the hair cell marker myosin VIIa. Some transplanted SCs engrafted in the cochlear basilar membrane
Chen 2018 [46]	Mouse *Slc26a4*-null	Human Induced pluripotent SC differentiated into otic epithelial progenitors and hair cell-like cells	Intracochlear (Scala tympani injection via RW)	Ciclosporin	daily injections intraperitoneally 500 mg/kg/day for 3 days	Transplanted SCs could migrate, engraft, and differentiate into hair cells Transplanted SCs in the organ of Corti formed synaptic connections with native spiral ganglion neurons
Schulze 2018 [47]	Guinea pig Normal hearing	Human Umbilical cord mesenchymal stromal cells	Intracochlear cochleostomy (scala tympani)	Ciclosporin	5.6 mg/kg/day	Only 2/6 specimens showed SC survival at 7 days
Lopez-Juarez 2019 [48]	Guinea pig Amikacin deafened (15 days, 400 mg/kg/day)	Human Otic progenitor cells from induced pluripotent stem cells	Intracochlear cochleostomy (scala tympani)	Ciclosporin (some animals NOT treated)	oral 15 mg/kg started 2 days before surgery and pursued until 7 days post-grafting	Human otic progenitor cells triggered an Immune response The number of surviving human OPCs was higher in ciclosporin-pre-treated animals Cell migration increased in ciclosporin-pre-treated animals
Eshraghi 2020 [49]	Rat Normal hearing	Rat Bone marrow mesenchymal stem cells	Intratympanic injection via pars flaccida	None	None	No induced oxidative stress No proinflammatory cytokines induced Caspase 3 pathway not activated Did not assess cell presence in cochlear
Chang 2020 [50]	Mouse Diphtheria toxin (DT) deafened DTR strain	Human Neural stem cell derived otic neuronal progenitors	Intracochlear (round window excision, to scala vestibuli and tympani)	None	None	No fibrosis or lymphocyte infiltration Some lymphocyte aggregation outside the membranous labyrinth Cell survival noted at 90 days

The assessment of the experimental risk of bias is displayed in Table 4. Notably, 100% of studies did not report randomisation of animals to treatment groups. Blinding at both intervention and assessment was generally either not reported or regarded as high risk. Overall, selection and performance bias were poorly accounted for in the included studies, while attrition and reporting bias were appraised as low risk.

**Table 4:** Graphical representation of the results from the SYRCLE risk of bias tool. Green indicates low risk of bias, yellow unclear and red indicates high risk of bias.

		Selection Bias			Performance Bias		Detection Bias		Attrition Bias	Reporting Bias	Other
Study	Reference	Sequence generation for randomisation	Groups similar at baseline	Allocation concealment	Random Housing	Blinding at intervention	Random outcome assessment	Blinding at assessment	Incomplete data outcome	Selective outcome reporting	Other sources of bias
Hildebrand et al. 2005	[40]	unclear	low	high	unclear	unclear	low	high	low	low	low
Lang et al. 2008	[41]	unclear	low	unclear	unclear	unclear	low	high	low	low	low
Cho et al. 2011	[42]	unclear	low	unclear	unclear	unclear	low	high	low	low	low
Zhang et al. 2013	[43]	unclear	low	unclear	unclear	unclear	low	high	low	low	low
Gokcan et al. 2016	[44]	unclear	low	unclear	unclear	unclear	low	high	low	low	low
Barboza et al. 2016	[45]	unclear	low	low	unclear	low	low	low	low	low	low
Chen et al. 2018	[46]	unclear	unclear	high	unclear	high	low	high	low	unclear	low
Schulze et al. 2018	[47]	unclear	unclear	high	unclear	high	low	high	low	unclear	low
Lopez-Juarez et al. 2019	[48]	unclear	low	unclear	unclear	high	low	high	low	low	low
Eshraghi et al. 2020	[49]	unclear	unclear	unclear	unclear	high	low	high	low	low	low
Chang et al. 2020	[50]	unclear	low	unclear	unclear	high	low	high	low	low	low

In vivo studies frequently did not report on the use or omission of immunosuppression, and reporting on the presence or absence of fibrosis or inflammatory cell infiltrate in the implanted cochlear has been variable. Stem cells varied in origin and the extent of differentiation occurring prior to *in vivo* use, and included mesenchymal stem cells. The route of administration of cells also varied; most cells were injected intracochlear with techniques such as cochleostomy (employed in 6/11 studies) potentially more traumatic and at risk of inflammatory response than round window injection via glass needle micropipette (3/11). One study used intratympanic delivery.

Importantly all studies employed a relatively short period before animal sacrifice and histological and immunohistochemical assessment of the cochlea. The potential for longer term, chronic rejection of transplanted cells has therefore not been investigated.

#### Choice of immunosuppressive agent

Ciclosporin has been the immunosuppressive agent of choice for cochlea stem cell transplant studies utilising immunosuppression, though the rationale for this choice is not elaborated in the identified clinical studies. Interestingly ciclosporin is one agent that has not previously been employed clinically for otological disease, and also has not been employed in any of the stem cell studies in allied areas.

Ciclosporin is a calcineurin inhibitor, supressing cell-mediated immune response through inhibiting production and release of lymphokines. It is clinically used to prevent allograft rejection, and is widely administered following solid organ transplant (kidney, liver, and heart). It has also attained common use in certain autoimmune conditions including psoriasis and rheumatoid arthritis. Ciclosporin is available for oral, intravenous or topical application (as eye drops). Monitoring is required (full blood count, renal function, liver function). In the short term mild adverse effects are common, and longer-term use is associated with increased risk of cardiovascular events and malignancies. Ciclosporin and other calcineurin inhibitors have been shown to exhibit minimal ototoxicity, though evidence is limited [51, 52].

#### Studies without immunosuppression

Five intracochlear stem cell studies did not use immunosuppression and investigated intracochlear inflammation [40, 42, 45, 49, 50]. Several authors examined cochlea histologically and found no evidence of inflammatory cell infiltrate [40, 42, 45]. Chang *et al*. noted fibrotic tissue infiltration in the scala vestibuli in some cochlea, and lymphocyte aggregation and dilated blood vessels in tissue adjacent to the transplanted cochlea, but there was minimal cell response within the membranous labyrinth [50].

Eshraghi *et al*. describe the most thorough evaluation of inflammatory response to stem cell transplantation [49], relating to the use of rat mesenchymal stem cells transplanted via an intratympanic injection. Comparing stem-cell injected cochlear to controls, they found no evidence of enhanced intracochlear oxidative stress (determined by 8-isoprostane immunostaining), no increased activation of the caspase3 pathway, and no increase in the generation of TNF-α, IL-1β, IL-6 and IL-12. It should be noted that cell type and route of delivery notably different to other studies and are potentially less likely to trigger an immune response. Notably, mesenchymal stem cells (MSCs) present low immunogenicity, and undifferentiated MSCs express low levels of human leukocyte antigen (HLA) class I and II molecules [53]. MSCs hinder T cells from the contact with antigen presenting cells [54] and fail to stimulate allogeneic peripheral blood mononuclear cells or T-cell proliferation [55].

No studies utilised autologously harvested mesenchymal cells, although this has been proposed as an approach to eliminate the need for immunosuppression to prevent rejection. Overall study heterogeneity was high, and there was no scope for meaningful comparisons of outcomes between studies grouped by use of immunosuppression.

#### Experimental studies on the role of immunosuppression

Only two studies have included methodology to experimentally assess the impact of immunosuppression in stem cell transplantation for hearing loss, and to date one has only been reported as a conference abstract. Lopez-Juarez *et al*. found that human otic progenitor cell (OPC) transplantation triggered an immune response in amikacin-exposed guinea pig cochlea [48]. They describe a moderate and limited inflammatory response after the injection human OPCs assessing cochlea up to 14 days post-graft. This reaction was assessed by the expression of IBA1, a marker of macrophages/microglia. IBA1-immunopositive cells appeared at day 4 post-transplantation in ototoxic-grafted cochleae, although the authors also note that IBA1-positive cells were upregulated in response to cochlear damage induced by ototoxicity, but this was more limited and focussed at the modiolus. In addition, double labelling revealed that some stem cells were immunopositive for IBA1, suggesting possible initiation of an immune response following OPC injection.

Lopez-Juarez *et al*. also found that the number of human OPCs and their migration increased in ciclosporin-pre-treated animals. In ciclosporin-treated animals, the distribution of labelled stem cells showed a gradient from base-to-apex with a higher number of labelled cells found at the basal and second cochlear turns in the ciclosporin treated. Labelled stem cells were also observed to migrate further from the basilar membrane, closer to the reticular lamina in the ciclosporin treated group. The authors conclude that transient and moderate immunosuppression may enhance the number of migrated human OPCs within the sensory epithelia.

Perhaps the most interesting study, which is only reported in abstract form, is by Hartmann *et al*. [56]. In this study the group appear to have transplanted otic progenitor cells to gerbils treated with ouabain to experimentally reduce spiral ganglion cells. Four different methods of immunosuppression were assessed. Two groups of gerbils were treated with single-agent systemic immunosuppression, either ciclosporin 15mg/kg subcutaneously daily or dexamethasone 40mg/kg subcutaneously weekly. The third group was treated with a combination of subcutaneous injection of ciclosporin and dexamethasone daily. The fourth group were treated with local immunosuppression alone in the form of a Gelitta sponge containing ciclosporin (5mg/0.1 ml) and dexamethasone (4mg/0.5ml) placed in the round window niche after stem cell injection. The authors report that preliminary results indicate that systemic immunosuppression is necessary to ensure successful transplantation of OPC, but no further details are given. The study authors were contacted for any updated data but failed to respond.

#### Summary

Several animal studies utilising stem cell treatment of the cochlea have not employed immunosuppression, and investigators did not detect evidence of an immune or inflammatory reaction within the cochlea. Many of these authors concluded that the cochlea displays immunological privilege that is sufficient to allow xenotransplantation [45, 49, 50]. Where immunosuppression has been used *in vivo*, systemic ciclosporin treatment has been used, and steroids, whether locally or systemically delivered, have not been investigated. In the only studies designed to explore the effect of immunosuppression, the study by Lopez-Juarez *et al*. and preliminary report by Hartmann *et al*., findings support a role for immunosuppression. Future work should explore the effect of different immunosuppressive agents, and care is required to isolate the inflammatory response promoted by the experimental model insult or stem cell introduction method from that of the injected cells themselves to determine the long-term requirement for immunosuppression.

## Discussion

This report draws on evidence relating to pharmacokinetic modelling, *in vitro* studies, animal and human clinical studies, in the context of both established clinical practices and early pre-human stem cell studies. There is considerable data relating to oral and intratympanic steroids as inner ear immunosuppressants, though much of the clinical data should be treated with caution as the pathophysiology and mechanism of steroid action in several conditions is poorly understood. The limitations of this review should be understood prior to arriving at clinical recommendations. Outcome one (preliminary review) should not be considered a comprehensive systematic review, but rather a preliminary search and discussion regarding cochlear immunosuppression in an attempt to assess the landscape prior to a rigorous systematic review for outcome two (primary systematic review). Due to the breadth of outcome one, it was not possible to acknowledge and discuss all included articles from the literature search, thus introducing bias of the authors. In contrast, outcome two presents a rigorous report of the literature. This review was not prospectively registered on PROSPERO.

Animal and human studies suggest intracochlear concentration and distribution of steroids and other drugs varies between systemic and local delivery routes, with some clinical data, primarily related to SSNHL, supportive of combination treatment including intratympanic therapy providing improved outcomes. The adverse effects of long-term systemic steroids are well established, but short course high dose oral steroid and intratympanic steroid delivery both have low risk profiles. Steroids have been commonly and successfully employed peri-transplant in human stem cell studies in allied fields, to suppress inflammation associated with the transplant procedure and acute rejection.

Non-steroid immunosuppression in the form of DMARDs and more recently biologics has been employed in autoimmune ear disease, but not typically other conditions. Delivered systemically or locally there is growing evidence for inner ear immunosuppression with these agents, in particular with a role in steroid-sparing treatment, or in those who are steroid-resistant. This has been supported by animal model studies, but contradictory findings on the extent of immunosuppression achieved are seen in both clinical and laboratory work.

Some cochlear regenerative stem cell studies in animal models did not employ immunosuppression, without evidence of rejection in the short term. In addition, some of the more recent stem cell studies in allied fields (largely RPE regeneration) have omitted immunosuppression, though are yet to report. However, two animal studies designed to investigate the role of immunosuppression have suggested it is important for grafted cell survival, and almost all allied stem cell studies have employed a robust immunosuppressive regimen. Given the early-phase nature of the planned cochlear regenerative studies immunosuppression therefore appears warranted to maximally avoid donor cell rejection.

From the findings of this review, it is apparent that steroid therapy may be beneficial in the peri-transplant period, via combined oral systemic and intratympanic administration routes.

It is also important to note that the majority stem cell trials identified in this report have employed immunosuppression including a calcineurin inhibitor; systemic tacrolimus for human stem cell trials in the fields of macular degeneration, ALS and spinal cord injury, and systemic ciclosporin for cochlear stem cell studies in animal models. There is also limited evidence that systemic ciclosporin is beneficial in terms of immune suppression and stem cell migration and survival in cochlear stem cell transplantation.

Based on the limited current evidence oral tacrolimus or ciclosporin could be recommended as immune suppression following cochlear stem cell transplantation. Systemically administered tacrolimus or ciclosporin is well-characterised and widely used clinically, although associated with a significant side effect profile. A recent large systematic review and metanalysis compared systemic tacrolimus and ciclosporin for immunosuppression following renal transplantation [57]. There was no statistical difference in graft loss or patient mortality, however fewer tacrolimus-treated patients had acute rejection at most time points, and this reached statistical significance at 6 months. In terms of adverse events, there was no significant difference between ciclosporin and tacrolimus for infection, but there was significantly less diabetes in patients treated with ciclosporin, and significantly less hypercholesterolaemia in tacrolimus treated patients at 1 and 5 years. Calculating the risk difference of each treatment: Treating 100 renal recipients with tacrolimus instead of ciclosporin would result in 1 less death, 1 less graft loss, 10 less patients with rejection, and 3 less hypercholesterolemia but 2 more patients would develop diabetes. An older (2005) Cochrane review found that tacrolimus was superior to ciclosporin in improving graft survival and preventing acute rejection after kidney transplantation, but increased post-transplant diabetes, neurological and gastrointestinal side effects [58]. The risk-benefit profile should be considered when selecting a calcineurin inhibitor–both of which have been employed in neural stem cell studies.

An alternative immunosuppressive agent would be a biologic agent as these have been shown in some studies to reduce intracochlear inflammation or improve outcomes in AIED. Basiliximab has been used in non-cochlear stem cell transplant immunosuppression, and like calcineurin inhibitors works via interleukin-2 inhibition. However, currently no data were identified regarding the use of biologics for immunosuppression following stem cell transplant, whether cochlear or in allied fields.

The above recommendations are based on well-established and proven immunosuppression regimens in clinical practice that could be implemented without further investigative work. However, further animal model work on the role of immunosuppression in stem cell transplants for hearing restoration is needed for a better understanding of both the timing and extent of inflammation post-transplant, and the extent to which acute and chronic rejection occurs. The extent of inflammation is also likely to be influenced by the technique of transplant, whether via RWM puncture, cochleostomy or involving further inner ear surgery. These techniques and/or the resulting inflammatory response may alter intratympanic and/or systemic drug delivery to the inner ear, possibly potentiating it. However, two areas require investigation; namely the potential benefits of continuous or high-frequency intratympanic drug delivery and an understanding of calcineurin-inhibitor inner ear drug delivery and pharmacokinetics.

Intratympanic drug delivery potentially greatly reduces the well-established risks of toxicity associated with systemic treatment using most immunosuppressants, whilst ensuring adequate dosing of the target organ. However, it still remains unclear what impact, if any, immunosuppression-related toxicity could have on cell survival, maturation, and function following transplantation [59].

In contrast to glucocorticoids, the inner ear pharmacokinetics of ciclosporin and tacrolimus have not been investigated. If considering the use of intratympanic ciclosporin or tacrolimus, Salt *et al*. have developed a simulation program for drug dispersion in the inner ear [60] that may be of value in selecting an intratympanic delivery regimen and drug concentration. They report that calculations are based on anatomical data (guinea pig, mouse, and human), and aim to enhance drug delivery through pharmacokinetic simulation. The software also includes information regarding all the fluid and tissue compartments of the cochlea (including spiral ligament, spiral ganglion, auditory nerve, etc.) and the fluid compartments of the vestibule. The software was the basis for calculations used in their two excellent reviews on SSNHL [30, 31].

As our understanding of both inner ear immunity and stem cell therapies improves, new targets for inner ear immune suppression will likely arise. Modulating cochlear macrophage function may not only allow manipulation of the immune response, but also an alteration in inner ear drug permeability and pharmacokinetics. Our understanding of regenerative cellular therapies and the associated need for immunosuppression is in its infancy, and the development of more targeted strategies to reduce immune-mediated rejection of transplanted stem cells is still required.

## Supporting information

S1 Checklist Completed PRISMA checklist for the manuscript. (DOCX)

S1 File Database search terms. (PDF)

S2 File Details of screened articles included those included and excluded. (CSV)
