# Supplementary material for: Current and emerging approaches to cochlear immunosuppression with translation to human inner ear stem cell therapy: A systematic review
Source: PLoS One. 2025 Feb 13;20(2):e0318165. doi: 10.1371/journal.pone.0318165 (PMC11825040; doi:10.1371/journal.pone.0318165)
Supplement: S1 File — (PDF) [file pone.0318165.s002.pdf]

S1 File: Database Search Terms

Ovid MEDLINE(R) and Epub Ahead of Print, In-Process, In-Data-Review & Other Non-Indexed Citations, Daily and Versions 1946 to January 6, 2023

(Cochle* or auditory or scala or perilymph or "inner ear*").ti,ab,kw. or cochlea/ or ear, inner/ or scala tympani/ or perilymph/
AND

(Immune or steroid* or dexamethasone or prednisone or prednisolone or Methylprednisone or methylprednisolone or hydrocortisone or glucocorticoid* or triamcinolone or Azathioprine or methotrexate or cyclophosphamide or Mercaptopurine or fluorouracil or DMARD or "Disease modifying anti-rheumatic drug" or eluting or "blood labyrinthine barrier" or BLB or "Auto-immune inner ear disease*" or "autoimmune inner ear disease*" or "stem cell*" or "precursor cell*").ti,ab,kw. or Stem Cells/ or steroids/ or dexamethasone/ or prednisone/ or prednisolone/ or methylprednisolone/ or hydrocortisone/ or glucocorticoids/ or triamcinolone/ or Azathioprine/ or methotrexate/ or cyclophosphamide/ or Mercaptopurine/ or fluorouracil/

Embase 1974 to 2023 January 10
(Cochle* or auditory or scala or perilymph or "inner ear*").ti,ab,kw. or cochlea/ or scala tympani/ or inner ear/ or perilymph/
AND

(Immune or steroid* or dexamethasone or prednisone or prednisolone or Methylprednisone or methylprednisolone or hydrocortisone or glucocorticoid* or triamcinolone or Azathioprine or methotrexate or cyclophosphamide or Mercaptopurine or fluorouracil or DMARD or "Disease modifying anti-rheumatic drug" or eluting or "blood labyrinthine barrier" or BLB or "Auto-immune inner ear disease*" or "autoimmune inner ear disease*" or "stem cell*" or "precursor cell*").ti,ab,kw. or steroid/ or dexamethasone/ or prednisone/ or prednisolone/ or methylprednisone/ or methylprednisolone/ or hydrocortisone/ or glucocorticoid/ or triamcinolone/ or Azathioprine/ or methotrexate/ or cyclophosphamide/ or Mercaptopurine/ or fluorouracil/ or Disease modifying anti-rheumatic drug/ or stem cell/

Web of Science (Core Collection)
TS=(Cochle* or auditory or scala or perilymph or "inner ear*")
AND

TS=(Immune or steroid* or dexamethasone or prednisone or prednisolone or Methyprednisone or methylprednisolone or hydrocortisone or glucocorticoid* or triamcinolone or Azathioprine or methotrexate or cyclophosphamide or Mercaptopurine or fluorouracil or DMARD or "Disease modifying anti-rheumatic drug" or eluting or "blood labyrinthine barrier" or BLB or "Auto-immune inner ear disease*" or "autoimmune inner ear disease*" or "stem cell*" or "precursor cell*")
Scopus
Title-abs-key (Cochle* or auditory or scala or perilymph or "inner ear*")
AND

Title-abs-key (Immune or steroid* or dexamethasone or prednisone or prednisolone or Methyprednisone or methylprednisolone or hydrocortisone or glucocorticoid* or triamcinolone or Azathioprine or methotrexate or cyclophosphamide or Mercaptopurine or fluorouracil or DMARD or "Disease modifying anti-rheumatic drug" or eluting or "blood labyrinthine barrier" or BLB or "Auto-immune inner ear disease*" or "autoimmune inner ear disease*" or "stem cell*" or "precursor cell*")

Results Table

Database	Results (18/03/2024)
Medline	2250
Embase	4035
Web of Science	2858
Scopus	5851

Results after deduplication: 8465
